# A Comprehensive Review of the Pharmacologic Management of Uterine Leiomyoma

**DOI:** 10.1155/2018/2414609

**Published:** 2018-01-28

**Authors:** Terrence D. Lewis, Minnie Malik, Joy Britten, Angelo Macapagal San Pablo, William H. Catherino

**Affiliations:** ^1^Program in Adult & Reproductive Endocrinology, Eunice Kennedy Shriver National Institute of Child Health and Human Development, National Institutes of Health (NIH), Bethesda, MD, USA; ^2^Department of Obstetrics and Gynecology, Uniformed Services University of the Health Sciences (USUHS), Bethesda, MD, USA; ^3^Department of Obstetrics and Gynecology, Walter Reed National Military Medical Center (WRNMMC), Bethesda, MD, USA

## Abstract

Uterine leiomyomata are the most common benign tumors of the gynecologic tract impacting up to 80% of women by 50 years of age. It is well established that these tumors are the leading cause for hysterectomy with an estimated total financial burden greater than $30 billion per year in the United States. However, for the woman who desires future fertility or is a poor surgical candidate, definitive management with hysterectomy is not an optimal management plan. Typical gynecologic symptoms of leiomyoma include infertility, abnormal uterine bleeding (AUB)/heavy menstrual bleeding (HMB) and/or intermenstrual bleeding (IMB) with resulting iron-deficiency anemia, pelvic pressure and pain, urinary incontinence, and dysmenorrhea. The morbidity caused by these tumors is directly attributable to increases in tumor burden. Interestingly, leiomyoma cells within a tumor do not rapidly proliferate, but rather the increase in tumor size is secondary to production of an excessive, stable, and aberrant extracellular matrix (ECM) made of disorganized collagens and proteoglycans. As a result, medical management should induce leiomyoma cells toward dissolution of the extracellular matrix, as well as halting or inhibiting cellular proliferation. Herein, we review the current literature regarding the medical management of uterine leiomyoma.

## 1. Introduction: Uterine Leiomyomata

Uterine leiomyomata, also referred to as myomas and fibroids, are the most common solid tumors of the gynecologic tract. These benign tumors are postulated to arise from a single, genetically altered, mesenchymal cell under the influence of gonadal hormones, namely, progesterone and 17*β*-estradiol. Epidemiologic data report that leiomyomata are virtually nonexistent prior to menarche and typically have an indolent course following menopause, strongly implicating gonadal hormones in the induction and maintenance of this disease process [1]. These benign tumors are found in 70% of women of European descent and more than 80% of women of African descent by 50 years of age [2–7]. Despite the high percentage of women affected by leiomyomata, it has been estimated that only 20%–30% will become symptomatic of their disease [8, 9]. Risk factors for the development of the disease have been identified and include increasing age, nulliparity, obesity, premenopausal status, personal history of hypertension, family history, race/ethnicity, time since last birth, and consumption of food additives and soybean milk [10, 11]. Of note, the strongest epidemiologic correlate is increasing age followed by a woman's race/ethnic background. To this end, women of African descent are at increased risk of developing multiple and larger leiomyomata at younger ages than their white counterparts [5].

Increasing tumor burden results in characteristic symptoms depending on the location of the tumor within the uterine corpus, that is, whether the tumor is submucosal, intramural, or subserosal. For example, leiomyomata distorting the uterine cavity (submucosal and intramural) often produce abnormal uterine bleeding (AUB), heavy menstrual bleeding (HMB), and/or intermenstrual bleeding (IMB) in the presence or absence of dysmenorrhea. These cavity-distorting tumors are often implicated in iron-deficiency anemia (secondary to AUB) and infertility. If a given patient is able to achieve pregnancy with a leiomyoma impacting the uterine cavity, they also are more likely to experience adverse pregnancy outcomes to include recurrent pregnancy loss (RPL), abnormal placentation (i.e., placenta previa), fetal malpresentation, preterm delivery, cesarean section, and postpartum hemorrhage [12–14]. Tumors in other locations, namely, intramural (well separated from the uterine cavity) and subserosal subtypes, are more often associated with pelvic pressure, pelvic pain, dyspareunia, chronic constipation, and urinary incontinence.

Hysterectomy is one of the most common surgeries performed on women and remains the only definitive treatment for leiomyomata. Myomectomy provides temporary reduction in uterine volume but is associated with a risk of recurrence estimated to be 11% with removal of a solitary fibroid and 26% or greater when multiple fibroids are removed over a 10–30-month time period [14, 15]. The failure of minimally invasive surgery to resolve disease contributes to more than 600,000 hysterectomies per year in the United States [16–18]. Estimates place the annual direct cost for surgery, hospital admissions, outpatient visits, and medications to be as high as $9.4 billion per year [19, 20]. However, when considering lost work time and hospital fees associated with poor obstetric outcomes, the economic burden of leiomyomata on the United States healthcare system is estimated to reach beyond $30 billion [20].

Multiple less invasive techniques including hysteroscopic myomectomy (used for submucosal fibroids), magnetic resonance imaging-guided focused ultrasound surgery, cryomyolysis, uterine artery embolization, and temporary occlusion of the uterine arteries have been employed to offer uterus-sparing options [21]. However, the safety and efficacy for use in women desiring future fertility have not been thoroughly defined for each of these less invasive procedures. In addition to the aforementioned therapeutic options, multiple medications have been employed to provide alternatives to hysterectomy. Despite the availability of such noninvasive therapies, current literature has not shown a definitive reduction in the numbers of hysterectomies performed in the United States given that most medical therapies result in a rapid return of symptoms and/or tumor volume with cessation of treatment [16, 17, 22, 23].

Because gonadal hormones induce, and maintain, leiomyoma growth via the production of an aberrant extracellular matrix (ECM), much research has focused on the development of medical agents to circumvent steroidal influence in an effort to reduce the burden of disease. These medications include centrally acting gonadotropin-releasing hormone analogs (leuprolide acetate, cetrorelix) and peripherally acting agents to include aromatase inhibitors, antiprogestins, and selective progesterone receptor modulators (SPRMs).

Taken together, the management of leiomyoma depends on given patient's symptoms, age, and desire for future fertility. In the case of women suffering from abnormal uterine bleeding, heavy menstrual bleeding, medical management with NSAIDs, progestin, combination of oral contraceptives, a levonorgestrel releasing intrauterine device, or tranexamic acid has been shown to be beneficial [24]. On the other hand, anatomic lesions causing abnormal uterine bleeding, such as uterine leiomyoma or polyps, may necessitate surgical intervention. Endometrial ablation and resection are minimally invasive surgical options to control abnormal uterine bleeding, heavy menstrual bleeding, in women with a normal uterine cavity who have completed childbearing. Women with small submucosal fibroids may also consider endometrial ablation for management of bothersome heavy menstrual bleeding. Despite the less invasive technique, patient satisfaction is not guaranteed. In fact, 27% of women who undergo endometrial ablation proceed with additional surgical interventions to include hysterectomy [17, 25]. Furthermore, patient symptoms and satisfaction are equally improved with the use of progesterone releasing intrauterine devices and endometrial ablation [26, 27].

For those women either not interested in definitive management with hysterectomy or considered to be poor surgical candidates, options for management of their disease should include agents aimed at reducing tumor burden by dissolution of the aberrant ECM. Herein, this manuscript will review the medical management of symptomatic uterine leiomyomata with particular emphasis placed on those medications that favor dissolution of the aberrant ECM.

## 2. Nonhormonal

### 2.1. Nonsteroidal Anti-Inflammatory Drugs (NSAIDs)

Nonsteroidal anti-inflammatory drugs (ibuprofen, naproxen, and mefenamic acid) have been employed in an effort to ameliorate abnormal uterine bleeding/heavy menstrual bleeding for a number of years. These agents inhibit the enzyme cyclooxygenase, which diminishes the production of prostaglandins. A Cochrane review evaluating the effectiveness of NSAIDs in the management of abnormal uterine bleeding/heavy menstrual bleeding included 18 studies [28]. The authors found the use of NSAIDs was superior to placebo but less effective than tranexamic acid, danazol, or the levonorgestrel releasing intrauterine device when evaluating the therapeutic impact on abnormal uterine bleeding [28]. Despite their usefulness with reducing both dysmenorrhea and blood loss, these agents have not been shown to lead to dissolution of the leiomyoma ECM.

### 2.2. Tranexamic Acid

Tranexamic acid is a synthetic lysine derivative that prevents fibrin degradation by competitively blocking lysine-binding sites on plasminogen, thereby preventing fibrin degradation. This action favors clotting, which reduces menstrual blood flow. Several randomized control trials have demonstrated a reduction in menstrual blood flow as compared to placebo [29–31]. Tranexamic acid was approved by the FDA in 2009 for the treatment of women suffering from abnormal uterine bleeding/heavy menstrual bleeding secondary to ovulatory disorders, not uterine leiomyoma.

Several studies have specifically evaluated the impact of tranexamic acid on women with symptomatic uterine leiomyomata [29–31]. In these studies, its utility in improving blood loss is not well established. Furthermore, these studies revealed an increased risk of necrosis and infarction of the leiomyoma, which could lead to pain and provide a potential site for infection. Despite the theoretical benefit in women with uterine leiomyomata, tranexamic acid has no effect on the ECM and reducing the burden of disease.

## 3. Hormonal Therapy

The steroid hormones 17*β*-estradiol and progesterone, in combination or progesterone only formulations, are commonly utilized to regulate heavy menstrual bleeding in women with and without uterine leiomyoma. Strict regulation of the menstrual cycle via these medications is particularly beneficial in women suffering from anovulation. Despite the aforementioned benefits, current evidence suggests medical therapies provide short-term relief, with many patients ultimately opting to pursue surgical therapies [32, 33].

### 3.1. Combined Oral Contraceptives

Given our current understanding of the importance of the gonadal hormones for initiation and continued growth of uterine leiomyomata, many physicians previously recommended against the use of such medications in women with uterine leiomyomata. Despite these fears, combined oral contraceptives have been utilized for women with leiomyomata and a meta-analysis found no association with progression of disease while using these medications [34]. In fact, this study found the risk of uterine leiomyomata associated morbidity was reduced by 17% in those who used combined oral contraceptives for 5 or more years.

The current data are limited regarding the effects of estrogen and progesterone. Estrogen and progesterone treatment, usually with combined oral contraceptive pills, may control abnormal uterine bleeding (by suppressing endometrial growth) and may not stimulate leiomyoma ECM.

### 3.2. Progesterone

Progesterone containing oral, injectable, and implantable contraceptives act to reduce blood loss by providing an inhibitory effect on endometrial cell proliferation leading to a thinner lining with less material to be shed during progestin withdrawal. However, as was the case with combined oral contraceptives, studies utilizing progesterone only contraceptives in the treatment of symptomatic uterine leiomyomata have demonstrated mixed results. To this point, there are studies in which the authors note reduction in leiomyomata size with progesterone only therapy, while others report an increase in leiomyomata size [35–38]. A well-designed, randomized controlled trial is needed to adequately study the effects of exogenous progestin in the treatment of women with uterine leiomyomata.

### 3.3. Levonorgestrel Releasing Intrauterine Device (LNG-IUD)

The levonorgestrel releasing intrauterine device (LNG-IUD) acts at the level of the endometrium to repress estrogenic stimulated growth thereby producing a thinned endometrial lining. In addition, there is virtually no uptake of levonorgestrel into the systemic circulation [33].

Progestin releasing intrauterine devices are effective at treating abnormal uterine bleeding associated with anovulation and is now approved by the Food and Drug Administration for this indication [33]. Small studies suggest the LNG-IUD may be effective for treatment of abnormal uterine bleeding/heavy menstrual bleeding in women with leiomyoma; however, no randomized controlled trials have been performed using this patient population [39].

## 4. Aromatase Inhibitors

Aromatase (CYP19) is an enzyme responsible for ovarian and peripheral conversion of androgens, namely, testosterone, to 17*β*-estradiol. Several* in vitro* studies revealed that uterine leiomyoma cells harbor intrinsic aromatase activity, thereby providing a direct source of steroid hormone to drive further growth through the development of an aberrant extracellular matrix [40]. This finding stimulated interest in the utilization of aromatase inhibitors as pharmacologic agents in the treatment of leiomyomata.

Based on their mechanism of action, aromatase inhibitors were hypothesized to have fewer side effects than the GnRH agonist, leuprolide acetate, with the benefit of a rapid effect. Several publications have shown reductions in leiomyomata volume and symptoms with the use of these agents [41–45].

One study using the aromatase inhibitor, CGS 20267, revealed the ability of that agent to inhibit ovarian and peripheral conversion of androgens to 17*β*-estradiol within 24 hours of first use [41]. A small open-label trial involving twenty patients evaluated the effects of a second aromatase inhibitor, anastrozole, on uterine volume without changes in circulating FSH or 17*β*-estradiol levels [46]. A subsequent randomized controlled trial compared letrozole, an aromatase inhibitor, against triptorelin, a GnRH analog [42]. The study ultimately found reductions (45% versus 33%, resp.) in tumor burden; however women in the letrozole-arm experienced fewer side effects and avoided symptoms associated with the initial GnRH flare.

A Cochrane review published in 2013 on aromatase inhibitors used for the management of uterine leiomyomas focused on one randomized control trial with 70 patients that met inclusion criteria [47]. The authors concluded there was insufficient evidence to support the use of aromatase inhibitors in the treatment of women with uterine fibroids [47]. In the absence of well-designed trials, these agents have yet to be approved by the United States Food and Drug Administration (FDA) for use in women with uterine leiomyomata.

## 5. Gonadotropin-Releasing Hormone Analogs

### 5.1. GnRH Agonist

In normal human physiology, sex steroid hormone production is a highly regulated process with major control centering at the hypothalamus, which is below the third ventricle and directly above the chiasma and pituitary gland. This gland exerts control over sex steroid production via the release of gonadotropin-releasing hormone (GnRH). When released in a specific, pulsatile fashion, GnRH induces the release of the pituitary gonadotropins Follicle Stimulating Hormone (FSH) and Luteinizing Hormone (LH), which in turn act at the level of the ovary to stimulate production of the sex-steroids, 17*β*-estradiol, and progesterone, respectively. Compounds that regulate ovarian stimulation and thereby decrease gonadal hormone production are attractive treatment options for women suffering from gonadal hormone-stimulated diseases such as leiomyomas.

As a class of medication, GnRH agonist (leuprolide acetate, goserelin acetate, and nafarelin acetate) has historically been considered the most effective presurgical therapy for symptomatic leiomyoma. They induce a premenarchal state notable for hypoestrogenism, by downregulation of the hypothalamic-pituitary-ovarian axis, amenorrhea, improvement in symptoms (namely, AUB-HMB/IMB), and rapid reduction in leiomyomata volume. That being said, the benefits achieved come with an unavoidable side effect profile to include vasomotor symptoms, vaginal dryness, sleep disturbances, myalgia, arthralgia, mood-swings, and potential cognitive impairment [33, 48, 49]. Long-term therapy, greater than 6 months, with GnRH agonists has been implicated in bone loss of approximately 6% [50]. Important to note, the benefits, and side effects, of GnRH agonists are temporary and reverse with the discontinuation of the medication [33, 49–52].

In one of the large scale clinical trials assessing leuprolide efficacy in women with symptomatic leiomyomata, 128 women were enrolled and placed into either the treatment or placebo arm [51]. Those in the treatment arm received 3.75 mg leuprolide acetate intramuscularly monthly for a total of 6 months. The authors found a 36% reduction in uterine volume at 12 weeks and 45% with 24 weeks of treatment. However, mean uterine volume returned to pretreatment size 24 weeks after cessation of leuprolide acetate [51].

Similar studies have been performed and produced similar results with all showing a 30–65% reduction of leiomyomata within 6 months of treatment with leuprolide acetate [51–55]. However, given the hypoestrogenic state and bone loss, most organizations including the American Congress of Obstetricians and Gynecologist (ACOG) recommend limiting the use of leuprolide acetate to symptomatic women scheduled to undergo surgery within 6 months of initiating therapy [33, 49]. If used longer, ACOG recommends that low-dose steroidal add-back therapy be considered to minimize continued bone loss and vasomotor symptoms. To this end, leuprolide acetate has been approved by the United States Food and Drug Administration for preoperative therapy in woman with iron-deficiency anemia secondary to leiomyomata.

On a molecular level, GnRH agonists decrease the expression of factors important for fibroid growth to include Transforming Growth Factor-Beta, Epidermal Growth Factor, and Insulin-like Growth Factor. Further data from our laboratory has shown reduction of the extracellular components collagen -1, fibronectin, and versican with leuprolide acetate treatment [56, 57].

### 5.2. GnRH Antagonist

Similar to the GnRH agonists, GnRH antagonists including cetrorelix acetate, ganirelix acetate, and Nal-Glu have been shown in clinical trials to reduce leiomyoma volume via induction of a hypoestrogenic state [58–62]. However, these medications are injected and must be taken every 1 to 4 days because there are currently no long-acting depot forms available in the United States, which limits their usefulness with regard to the medical treatment of leiomyomata.

### 5.3. Antiprogestins

Progesterone receptor A and B (PR-A, PR-B) protein has been shown to be elevated within leiomyomata, as compared against adjacent myometrium [63, 64]. Additional publications have directly implicated progesterone action via PR-A and PR-B with the production of an aberrant extracellular matrix. Taken together, these points make inhibitors of the PR an area of interest for the medical management of leiomyomata.

Mifepristone, also known as RU 38486 & RU486, is the most extensively studied progesterone receptor antagonist in leiomyomata [65–69]. This compound is a competitive antagonist and has higher infinity for the ligand-binding domain of the PR than does progesterone [70, 71]. Several studies have shown mifepristone is capable of improving symptoms and reducing the volume of leiomyomata [65, 66, 72–74]. One study compared increasing doses of mifepristone in 40 premenopausal women with symptomatic leiomyomata [66]. Study participants took either 5 or 10 mg of mifepristone daily for one year and were followed with serial imaging. Ultimately, the authors found a reduction in uterine volume of 48% after 6 months of treatment and 52% after one year. Amenorrhea occurred in 65% at 6 months and 70% at one year. Although encouraging, there were 6 cases of simple endometrial hyperplasia without atypia in the 10 mg group. A subset of study participants was followed for an average of 6 months at the completion of the study and most had modest reenlargement of their leiomyomata.

A Cochrane review evaluated the usefulness of mifepristone for symptomatic leiomyomata [72]. Of all studies on this topic, 3 randomized controlled trials with a total of 112 patients with symptomatic leiomyomata met inclusion criteria [65, 73, 74]. The review concluded mifepristone indeed reduced abnormal uterine bleeding-heavy menstrual bleeding/intermenstrual bleeding, and also improved fibroid-specific quality of life. Despite the aforementioned improvements, the Cochran review found no significant reduction in leiomyomata volume with mifepristone therapy.

Overall, the weight of the evidence suggests that treatment with mifepristone leads to a reduction in patient symptoms associated with leiomyomata that is comparable to GnRH analogs without detrimental effects on bone mineral density. However, given the unopposed estrogen stimulation of the endometrium with development of endometrial hyperplasia, caution must be taken given the potential for development of estrogen-dependent endometrial cancers. It is for this reason that approval has not been sought from the Food and Drug Administration for use of these medications in women with symptomatic leiomyomata.

## 6. Selective Progesterone Receptor Modulators (SPRMs)

Selective progesterone receptor modulators (SPRMs) are a class of medications that are structurally similar to the antiprogestin mifepristone. Similar to the more widely known Selective Estrogen Receptor Modulators (SERMs), SPRMs have tissue-specific agonist and antagonist effects making them prime agents for use in the treatment of uterine leiomyomata. Members of this class of medication include telapristone acetate (also known as CDB-4124), asoprisnil (also known as J867), and ulipristal acetate (also known as CDB-2914).

Asoprisnil (J867) was originally developed by Schering and TAP Pharmaceutical Products in the mid to late 1990s [75]. The major metabolite of the drug, J912, was shown to have high-binding affinity for the progesterone receptor, moderate affinity for glucocorticoid receptor, low affinity for androgen receptor, and no binding affinity for estrogen or mineralocorticoid receptors. Because of its promising* in *vitro and animal work, the drug went to clinical trial for treatment of symptomatic uterine fibroids in humans. These* in vitro *models have shown that asoprisnil downregulates growth factors and synthesis of collagens, which are important in increasing leiomyoma, bulk [76, 77]. Despite these promising attributes, phase III clinical trials were halted secondary to concerning progesterone receptor-modulator associated endometrial changes (PAECs). The histologic changes associated with PAECs are dilated, weakly secretory endometrial glands with mitotic figures, and stromal effects ranging from compaction to nonuniform edema [78–80]. A panel of gynecologic pathologists examined these changes and concluded they should not be considered a safety concern [81].

Telapristone acetate (CDB-4124) was developed at the National Institutes of Health, Contraception Development Branch [82]. The phase III, open-label, parallel, randomized, multicenter study was halted in 2009 after patients were found to have significant elevations in their liver function tests (LFTs), suggesting hepatotoxicity.

Similar to telapristone acetate, ulipristal acetate (CDB-2914) was developed by the National Institutes of Health, Contraception Development Branch. This drug was made available as an emergency form of contraception in Europe in 2009 and subsequently approved in the United States in 2010 [83]. In this capacity, 30 mg of ulipristal acetate has been shown to be effective up to 5 days after unprotected intercourse [84].

Given the enthusiasm for the use of SPRMs in the management of leiomyoma, a group at the National Institutes of Health (NIH), National Institute of Child Health and Disease Branch (NICHD), performed a randomized control trial including 22 premenopausal women with symptomatic uterine leiomyomata [85, 86]. Women were assigned into one of three arms: CDB-2914 10 mg, CDB-2914 20 mg, or placebo for the equivalent of 3 menstrual cycles. The authors found a significant reduction in leiomyomata volume by an average of 21% (10 mg arm) and 36% (20 mg arm) after 3 months of treatment. Moreover, patients experienced an improvement in symptoms based on the Uterine Fibroid Symptom Quality of Life assessment. The same group published a second, randomized, double-blind, placebo-controlled, phase IIb study in 42 premenopausal women in which symptomatic uterine leiomyomata were randomized to receive placebo, CBD-2914 10 mg, or 20 mg for 12 weeks (treatment 1). A second 12-week treatment with CDB-2914 was offered. Again, the authors found significant improvements in abnormal uterine bleeding, quality of life, and leiomyomata volume at 3 and 6 months of treatment [86].

Subsequent to the NIH trials, a European group evaluated the ability of ulipristal acetate to reduce symptoms and tumor burden in symptomatic uterine leiomyomata. The PGL4001 (ulipristal acetate) Efficacy Assessment in Reduction of symptoms due to uterine Leiomyoma (PEARL) I trial compared ulipristal acetate to placebo in the preoperative management of 242 premenopausal women suffering from symptomatic leiomyomata [87]. The authors concluded that treatment with ulipristal acetate for 13 weeks controlled bleeding in 91% of women receiving 5 mg, 92% of women receiving 10 mg, as compared to 19% of those receiving placebo. There was 21% and 12% reduction of leiomyomata volume in those receiving 5 mg and 10 mg, respectively.

The authors of the PEARL I trial then performed a noninferiority trial comparing ulipristal acetate (5 mg and 10 mg) to leuprolide acetate [88]. The study included 307 premenopausal women with symptomatic leiomyomata. Following a 3-month treatment 90% of patients in the 5 mg arm and 98% in the 10 mg arm had control of bleeding, as compared to 89% in the leuprolide acetate group. Leiomyomata volume was found to have decreased by 36%, 42%, and 53%, respectively.

The leaders of the European group subsequently performed a third clinical trial with repeated intermittent open-label UPA courses, each followed by randomized double-blind norethisterone acetate (NETA) or placebo including 291 premenopausal women with symptomatic leiomyomata. The authors found the median fibroid volume change from baseline was −63%, −67%, and −72% after treatment courses 2, 3, and 4, respectively. The authors conclude that repeat courses of ulipristal acetate control symptoms and significantly reduce leiomyoma volume [89].

Taken together, the PEARL trials provided sufficient evidence for the regulatory agencies of the European Union to approve ulipristal acetate as a preoperative treatment of symptomatic leiomyomata. To date, there are insufficient studies in the United States and therefore the Food and Drug Administration has not approved ulipristal for use in symptomatic uterine leiomyoma.

Similar to antiprogestins that inhibit progesterone activity in the endometrium, leading to unopposed estrogen stimulation with the potential development of endometrial intraepithelial neoplasia leading to Type I endometrial cancers, many have concerns regarding the long-term treatment of patients with ulipristal acetate. In one early study evaluating CBD-2914 as a contraceptive, 56 normally cycling women were treated with a single dose of ulipristal acetate (10 mg, 50 mg, or 100 mg) or placebo within 48 hours of ovulation and followed with transvaginal ultrasound (to evaluate endometrial thickness) and endometrial sampling (to evaluate potential changes within the endometrium). Of the 56 endometrial biopsies performed, one patient who received 50 mg of ulipristal acetate was noted to have findings consistent with benign endometrial intraepithelial neoplasia. Repeat luteal phase endometrial sampling performed two months later was negative for pathologic endometrial changes [90]. A single case report discussed a patient with benign metastatic leiomyoma who was treated with ulipristal acetate for 5 years continuously. The authors performed regular endometrial sampling over the patient's treatment course and found no evidence of endometrial intraepithelial neoplasia or progesterone receptor-modulator associated changes (PRACs), suggesting long-term therapy with this ulipristal acetate may be safe from an endometrial standpoint. A more recent systematic review evaluating the effects of SPRMs on the endometrium evaluated 1450 treated with ulipristal acetate [91]. The authors found 6 cases of endometrial pathology in women undergoing or previously treated with ulipristal acetate. Five women were diagnosed with benign endometrial intraepithelial neoplasia (previously called simple or complex hyperplasia without atypia) and one case of endometrial intraepithelial neoplasia (formerly referred to as simple or complex hyperplasia with atypia). The single case of EIN resolved spontaneously during treatment. The authors found no cases of endometrial cancer during or after treatment with ulipristal.

**Figure 1:**
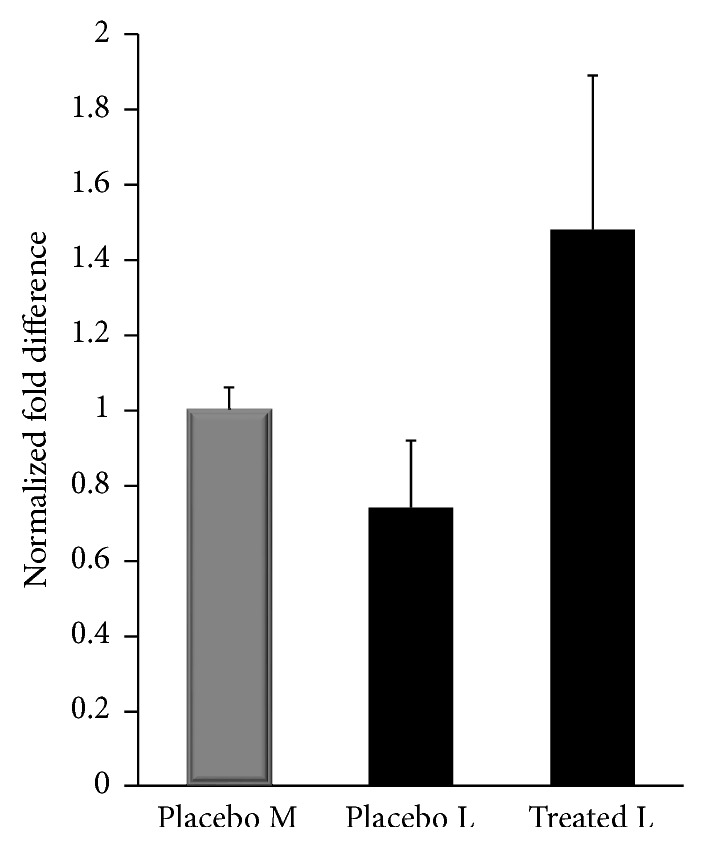
Fibrillin mRNA transcripts in placebo and ulipristal acetate treated patient samples.

**Figure 2:**
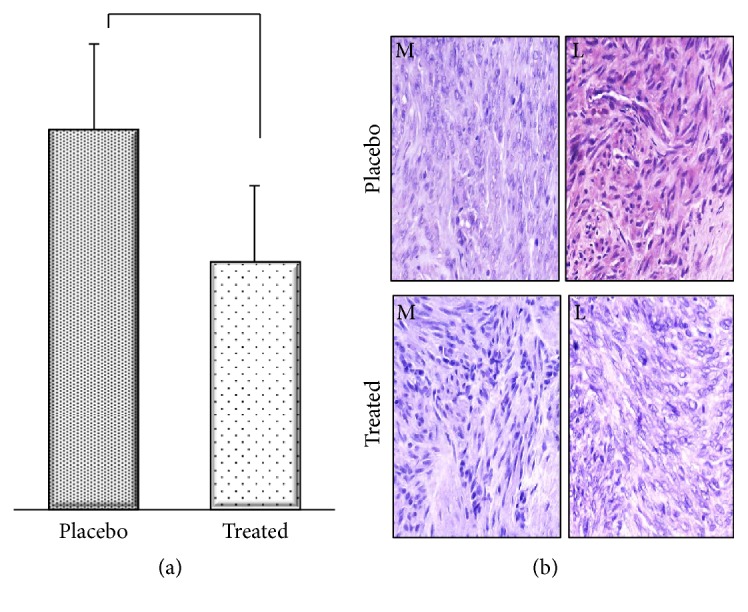
(a) Immunohistochemistry evaluation of TGF-*β*3 expression in patient matched myometrium (M) and leiomyoma (L) representative specimen. (b) Quantitation of TGF-*β*3 immunostaining revealing a decrease in TGF-*β*3 expression in treated leiomyoma, as compared to placebo.

Our laboratory performed an RNA sequence (RNAseq) analysis on placebo and ulipristal acetate treated patient matched leiomyoma and normal myometrium samples from the ulipristal (CBD-2914) clinical trials in an effort to identify novel pathways involved in the UPA-dependent reduction of uterine leiomyoma [85, 86]. Interesting observations from this set of experiments were alterations in the profibrotic, Transforming Growth Factor-*β*3 (TGF-*β*3) signaling pathway. First, the RNAseq data revealed a marked increase in Fibrillin transcripts in UPA-treated leiomyoma, as compared to placebo (Figure 1). Fibrillin is known to attenuate TGF-*β*3 signaling. Furthermore, there was a reduction in TGFRI, TGFRII, and TGF-*β*3 transcripts in UPA-treated leiomyoma. These findings were confirmed in proteomic studies utilizing both Western immunoblotting on proteins extracted from the study tissue and also immunohistochemistry on tissue (Figure 2).

The most significant changes in transcript levels were seen in those coding for the proteoglycan, versican (VCAN). This molecule is negatively charged, with large numbers of hydrophilic glycan components that form high-molecular weight aggregates with hyaluronic acid. Because of its structure, VCAN promotes hydrostatic pressure within the interstitial space. Therefore, reductions in VCAN would, in theory, lead to dehydration of uterine leiomyoma. Our analysis of the RNAseq study revealed VCAN transcripts were 4–6 times higher in leiomyoma than surrounding myometrium (data not shown). Furthermore, mRNA transcripts and protein expression were reduced in UPA-treated leiomyoma, which is consistent with the rapid reduction in leiomyoma volume seen in the CDB and PEARL clinical trials.

Taken together, the clinical and laboratory data on ulipristal acetate suggests it may act via the rapid induction/alteration of osmoregulatory genes to change osmotic forces leading to an initial rapid reduction in leiomyoma volume over the first 3 months of treatment followed by a slower reduction in leiomyoma volume [92]. Work recently presented by Donnez and Dolmans reports that leiomyoma can be completely resolved with repeated doses of UPA, suggesting this medication may have more than one mechanism by which it acts to reduce leiomyoma. Evaluation of uterine leiomyoma treated longer than 3 months may be required to fully elucidate the mechanisms by which this medication exerts its effects.

## 7. Conclusions

Management of symptomatic uterine leiomyomata must be individually tailored to patient symptoms, desires for future fertility, age, and location of the leiomyoma. A list of the medications reviewed herein is included within Table 1.

The GnRH agonist, leuprolide acetate, had been considered to be superior to any other medication for reduction of symptoms and tumor burden. However, the side effect profile (vasomotor symptoms, vaginal dryness, potential cognitive impairment, bone loss associated with long-term use, and rebound of uterine volume with discontinuation) limits the usefulness of this class of medications. To circumvent several of the side effects of GnRH analogs, practitioners have employed hormone add-back therapy with good success.

The use of combined oral contraceptive pills and progesterone only formulations has demonstrated benefit in treating abnormal uterine bleeding and dysmenorrhea without definitive proof of either reduction or enhancement of uterine leiomyoma volume. Well-designed, randomized control trials should be performed to better elucidate the utility of hormonal medications in the long-term management of uterine leiomyoma.

Given the prevalence, and morbidity, associated with uterine leiomyoma the promise of a long-term medical solution is encouraging with the advent of selective progesterone receptor modulators, most notably ulipristal acetate. This medication has been approved by the European regulatory agencies for use as a preoperative agent in women with symptomatic leiomyomata and anemia. Further work using this agent has shown complete resolution of uterine leiomyomata with repeated courses. There had been concern for endometrial pathology given the mechanism of action of the drug, but to this end the risk of development of endometrial hyperplasia or Type I endometrial carcinoma appears to be very low. That being said, it is currently still advisable that treatment with this agent be followed closely for evidence of endometrial pathology.

## Figures and Tables

**Table 1:** Medical management of symptomatic uterine fibroids.

	Dosing	Tx reduces leiomyoma volume?	Used to treat leiomyoma in the US?
*Nonsteroidal anti-inflammatory drugs (NSAIDs)*			
Ibuprofen	600 mg orally daily starting on the first day of menstruation	No	Yes
Mefenamic acid	500 mg orally three times per day starting on the first day of menstruation	No	Yes
Naproxen	500 mg by mouth twice daily starting on the first day of menstruation	No	Yes

*Antifibrinolytics*			
Tranexamic acid	1.3 g orally three times per day for 5 days	No	Yes
	10 mg/kg iv (maximum 600 mg/dose) every 8 hours	No	Yes

*Combined contraceptives*			
Oral, transdermal		No	Yes
Cyclic or noncyclic			

*Progestin-only therapies*			
Norethindrone-contraceptive pills	0.35 mg by mouth daily	No	Yes
Levonorgestrel releasing intrauterine device (IUD)	Intrauterine placement by healthcare professional; lasts 3–5 years depending on the device	No	Yes
Medroxyprogesterone (MPA)	Depo 150 mg intramuscularly every 12 weeks	No	Yes
	2.5–10 mg orally 12–14 days/month		

*Aromatase inhibitors*			
Letrozole	2.5 mg orally for 12 weeks	Insufficient evidence	No

*Gonadotropin-releasing hormone (GnRH) analogs*			
*GnRH agonists*			
Leuprolide acetate	Depot 7.5 mg intramuscularly every month	Yes (30–65%), reversible	Yes
	Depot 22.5 mg intramuscularly every 3 months		
	Depot 30 mg IM every 4 months		
	Depot 45 mg IM every 6 months		
	Eligard: 7.5 mg subcutaneously (sq) monthly/22.5 mg sq every 3 months/30 mg every 4 months/45 mg sq every 6 months		No
	Leuprolide acetate: 1 mg sq daily		No

*GnRH antagonists*			
Cetrorelix	3 mg sq every 4 days	Yes, reversible	No
	Depot 60 mg sq on cycle day 2		

*Antiprogestins*			
Mifepristone	5–50 mg orally daily for 3–12 months	Insufficient evidence	No

*Selective Progesterone Receptor Modulators*:			
Ulipristal acetate	10–20 mg po daily for 3 months	Yes (12–53%), appears to be a stable reduction	No^*∗*^

^*∗*^Approved for the presurgical treatment of symptomatic uterine leiomyoma in the European Union.
